# Supplementary material for: Insight into Hyper-Branched Aluminum Phosphonate in Combination with Multiple Phosphorus Synergies for Fire-Safe Epoxy Resin Composites
Source: Polymers (Basel). 2020 Jan 1;12(1):64. doi: 10.3390/polym12010064 (PMC7023559; doi:10.3390/polym12010064)
Supplement: Supplementary file 1 [file polymers-12-00064-s001.pdf]

polymers

MDPI

Supplementary Information

Insight into Hyper-Branched Aluminum Phosphonate in Combination with Multiple Phosphorus Synergies for Fire-Safe Epoxy Resin Composites

Yao Yuan [1], Bin Yu [2], Yongqian Shi [3], Long Mao [1], Jianda Xie [1], Haifeng Pan [4], Yuejun Liu [1,*] and Wei Wang [5,*]

[1] Fujian Provincial Key Laboratory of Functional Materials and Applications, School of Materials Science and Engineering, Xiamen University of Technology, Xiamen 361024, People's Republic of China; yuanyao@mail.ustc.edu.cn (Y.Y.); maolong0412@163.com (L.M.); xiejianda@xmut.edu.cn (J.X.)

[2] Centre for Future Materials, University of Southern Queensland, Toowoomba, QLD 4350, Australia; ahu07yb@gmail.com

[3] College of Environment and Resources, Fuzhou University, Fuzhou 350002, People's Republic of China; shiyq1986@fzu.edu.cn

[4] Faculty of Engineering, China University of Geosciences (Wuhan), Wuhan, Hubei, 430074, People's Republic of China; hfpan19@163.com

[5] State Key Laboratory of Fire Science, University of Science and Technology of China, Hefei, 230026, People's Republic of China

* Correspondence: wwei433@mail.ustc.edu.cn (W.W.); yjliu_2005@126.com (Y.L.)

Characterization

^1H and ^{31}P nuclear magnetic resonance (^1H and ^{31}P NMR) spectra were recorded on an AVANCE 400 Bruker spectrometer at room temperature using DMSO-d and D_2O as the solvent, respectively.

Fourier transform infrared (FTIR) spectra were obtained by a Nicolet 6700 spectrometer (Nicolet Instrument Company, USA) using KBr pellets. The wavenumber range was 400–4000 cm^{-1} and the resolution was 4 cm^{-1}.

The crystal-phase properties of the samples were analyzed with a powder X-ray diffractometer (XRD) (Japan Rigaku D Max-Ra) using a rotating anode X-ray diffractometer equipped with a Ni filtered Cu-Ka tube (λ = 1.54178 Å) in the 2θ range from 10° to 70° with a scanning rate of 4 min^{-1}.

The thermal stability was determined by thermogravimetric analysis (TGA), which was performed using a Q5000IR (TA Instruments) thermo-analyzer instrument at a linear heating rate of 20 °C/min from room temperature to 800 °C under nitrogen atmosphere.

Limiting oxygen index (LOI test was conducted using a HC-2 oxygen index meter (LOI analysis instrument company, Jiangning, China) according to ASTM D2863. Size of the specimens for the measurement was 100 × 6.5 × 3.0 mm^3.

Vertical burning UL-94 test was performed on a CFZ-II horizontal and vertical burning tester (Jiangning Analysis Instrument Co., China) with the sample dimension of 127 × 12.7 × 3 mm^3 according to ASTM D3801.

The combustion behaviors of the control EP and its composites were tested with a cone calorimeter (Stanton Redcroft, UK) according to ISO 5660 standard. Each specimen with size of 100 × 100 × 3 mm^3 was wrapped with an aluminum foil and burned at an external heat flux of 35 kW/m^2.

Real-time Fourier transform infrared spectroscopy (RT-FTIR) was used to study the thermo-oxidative degradation of samples. The FTIR spectra were recorded in the range of room temperature to 600 °C at a heating rate of 10 °C/min under air on a MAGNA-IR 750 spectrometer (Nicolet Instrument Company, USA).

Thermogravimetric-Fourier transform infrared spectroscopy (TG-FTIR) was conducted with a TGA Q5000 thermogravimetric analyzer, coupled with a Nicolet 6700 spectrophotometer by a stainless steel transfer pipe. Thermal analyzer was conducted in the range from room temperature to 800 °C with a heating rate of 20 °C /min under inert condition.

The morphology of flame retardant and the char layers of the samples after cone test were investigated by using scanning electron microscope (SEM; KYKY1010B, Shanghai Electron Optical Technology Institute, China). The samples were coated with gold/palladium alloy.

Raman spectra of the char residues were obtained using a SPEX-1403 laser Raman spectrometer (SPEX Co., USA) from 500 to 2000 cm^{-1}.

Table S1. Assignments of the peaks in FTIR spectra of cured epoxy resin and its composites.

FTIR (cm^{-1})	Assignments
3412	Stretching vibration of O-H groups
2875–2966	Stretching vibrations of $-CH_2$ and $-CH_3$
1090	Stretching vibration of P-O-C
1603	Stretching vibration of C=C
1110	O-H bending vibration
1502	Fingerprint (C-O stretching vibration, $-CH_3$, $-CH_2-$ and $-CMe_2-$ deformation vibration)
1250	Stretching vibration of P=O
1090	Stretching vibration of O=P-O
820,747	C-H bending, rocking vibration

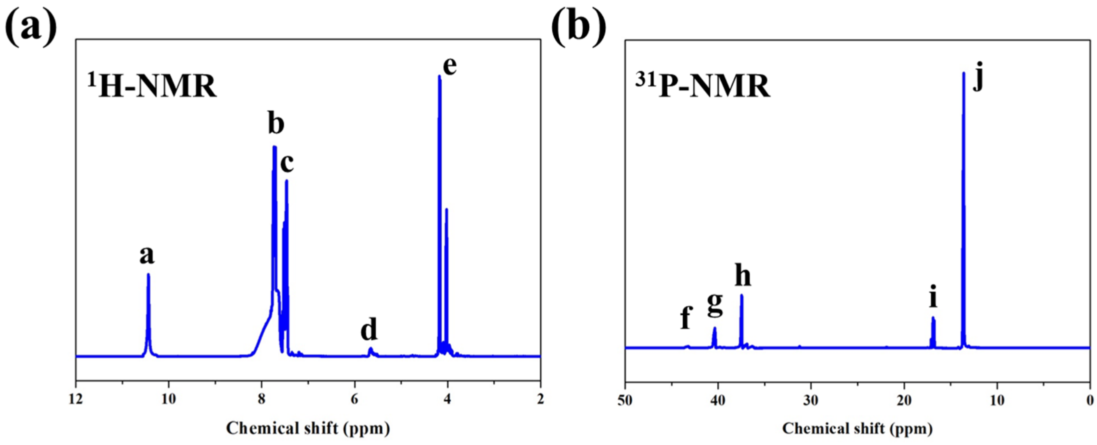

Figure S1. The ^{1}H NMR spectrum (**a**) and ^{31}P NMR spectrum (**b**) of HPP with DMSO-d6 as the solvent.

.

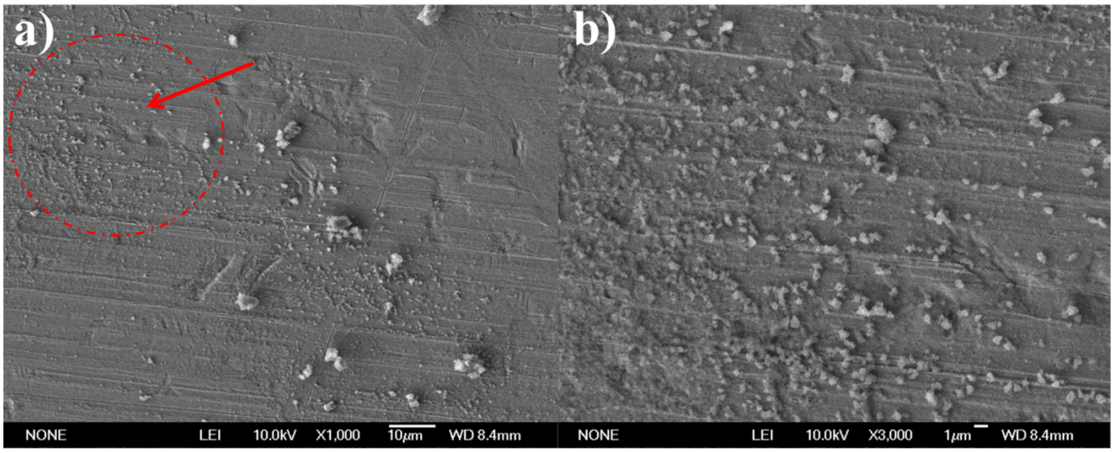

Figure S2. SEM observations of submicro-scaled AHPP: low-magnification (**a**) and magnified image (**b**).

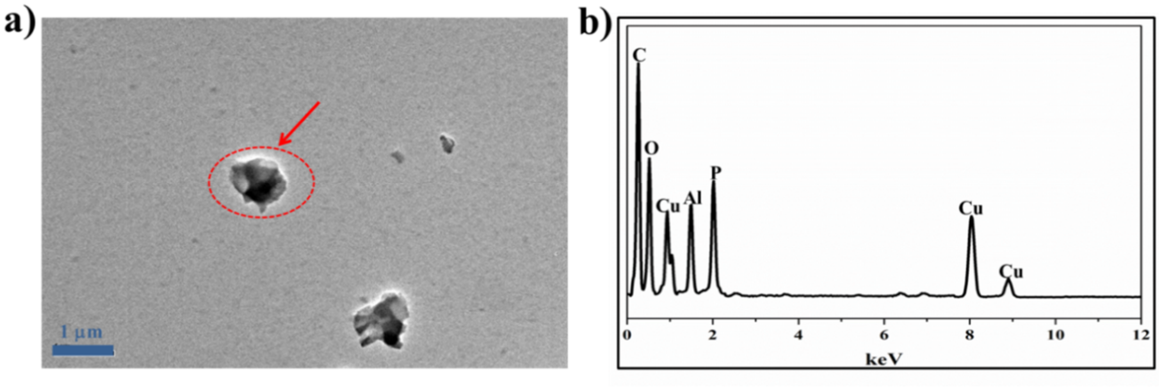

Figure S3. TEM image (**a**) and EDX spectrum (**b**) of submicro-scaled AHPP.

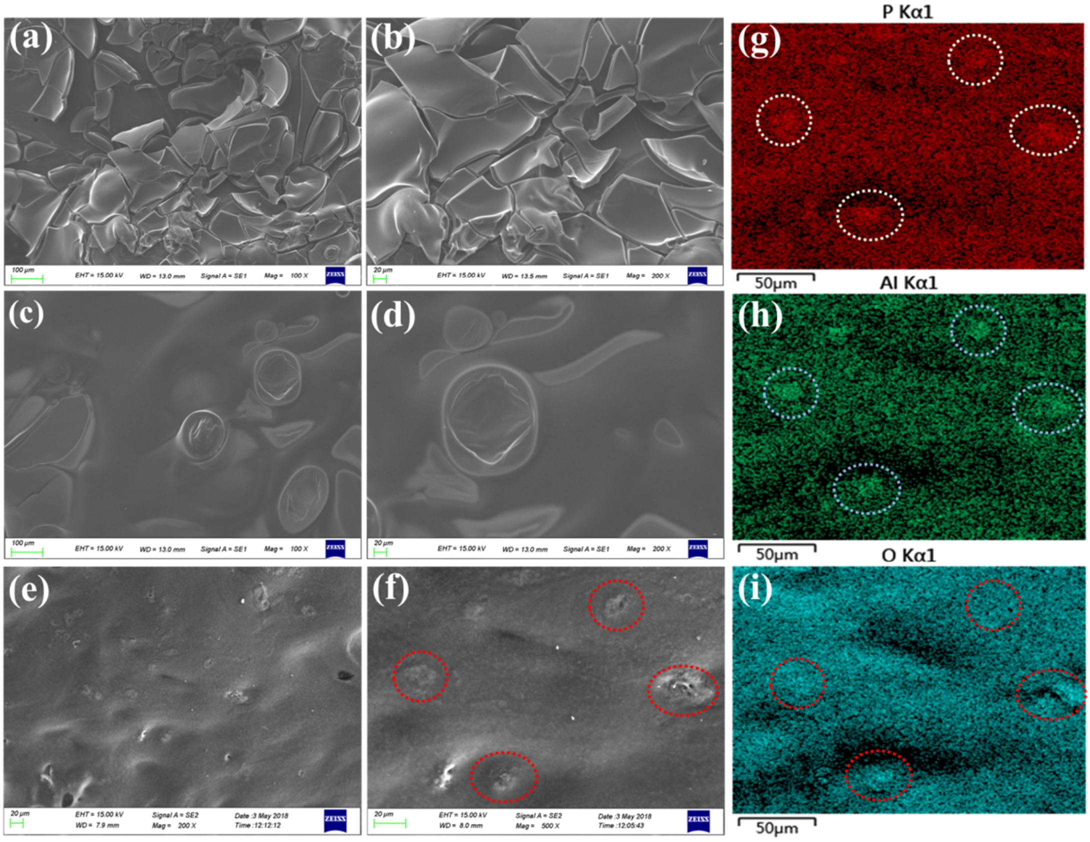

Figure S4. SEM micrographs of the char residue of pristine EP (**a**,**b**), EP/DOPO-AHPP (**c**,**d**), and EP-AHPP (**e**,**f**), and elemental distribution from EDX mapping of the EP-AHPP composite (**g**–**i**).
